# Development of a core outcome set for cardiovascular diabetology: a methodological framework

**DOI:** 10.3389/fendo.2023.1271891

**Published:** 2023-12-06

**Authors:** Jiao Jiao, Lingmin Chen, Yong Peng, Qingyi Jia, Ying He, Yonggang Zhang, Nian Li

**Affiliations:** ^1^ Department of Anesthesiology and National Clinical Research Center for Geriatrics, West China Hospital, Sichuan University and The Research Units of West China, Chinese Academy of Medical Sciences, Chengdu, China; ^2^ Department of Cardiology, West China Hospital, Sichuan University, Chengdu, China; ^3^ Department of Endocrinology and Metabolism, West China Hospital, Sichuan University, Chengdu, China; ^4^ Department of Integrated Traditional and Western Medicine, West China Hospital, Sichuan University, Chengdu, China; ^5^ Chinese Evidence-based Medicine Center, West China Hospital, Sichuan University, Chengdu, China; ^6^ Department of Periodical Press and National Clinical Research Center for Geriatrics, West China Hospital, Sichuan University, Chengdu, China; ^7^ Nursing Key Laboratory of Sichuan Province, West China Hospital of Sichuan University, Chengdu, China; ^8^ Department of Medical Administration, West China Hospital, Sichuan University, Chengdu, China

**Keywords:** cardiovascular diabetology, consensus, core outcome set, efficacy, safety

## Abstract

**Background:**

Cardiovascular diabetology is an emergent field focusing on all aspects of diabetes/cardiovascular interrelationship and metabolic syndrome. High-quality evidence needs to be provided to determine the efficacy and safety of interventions in cardiovascular diabetology. The heterogeneity of outcomes among trials limits the comparison of results, and some outcomes are not always meaningful to end-users. The cardiovascular diabetology core outcome set (COS) study aims to develop a COS of interventions for cardiovascular diabetology. In this paper, we introduce the methodological framework for developing the COS.

**Methods:**

The COS development will include the following steps: (a) establish the COS groups of stakeholders, including international steering committee, Delphi survey group, and consensus meeting group; (b) systematic reviews of outcomes used in trials of cardiovascular diabetology; (c) semistructured interview of stakeholders for outcomes of cardiovascular diabetology; (d) generate a list of candidate outcomes and determine the original outcome pool; (e) Delphi survey with stakeholders of cardiovascular diabetology to select potential core outcomes; and (f) review and endorse the cardiovascular diabetology COS by expert consensus meeting.

**Conclusions:**

This current study reports the methodological framework to develop a COS in cardiovascular diabetology and will provide evidence for the future development of COS in cardiovascular diabetology.

## 1 Introduction

Cardiovascular diabetology is an emergent field that focuses on all aspects of diabetes/cardiovascular interrelationship and metabolic syndrome (1–3). It includes research from clinical, genetic, experimental, pharmacological, epidemiological, and molecular biology fields and is mainly based on the combination of endocrinology and metabolism with cardiology (4–8). The objective of clinical research in the field is to help manage diabetes, promote cardiovascular health, reduce cardiovascular adverse events, and improve the long-term prognosis (9–13). The research mainly involves drug treatment and management (12), lifestyle intervention (14), and other aspects.

Performing high-quality trials is the key to effectively produce relevant evidence (15). Developing a high-quality systematic review is of great value to the disease’s diagnosis, treatment, and prevention (16). Therefore, trials have been published in cardiovascular diabetology (2, 10–12). However, because the results of cardiovascular diabetes are complicated, the reported outcomes are also different in trials (2, 10–12). In some trials, cardiovascular events were subjectively reported by the patients, and insufficient and under-reported problems always existed (1, 12). These nonstandard problems of outcomes will result in the difficulty of comparing and merging existing results in the systematic review, wasting resources, and limiting the synthesis of future evidence (17, 18).

In the cardiovascular diabetology field, if the outcomes are safety outcomes, there are even more differences (18). How to solve the nonstandardized reporting of outcomes and improve the application and dissemination of results is another challenge (19, 20). Previous studies suggested using the terminology set of ADR codes to standardize and help the reporting of safety outcomes (21). Some studies also suggested standardizing outcomes of trials by developing a core outcome set (COS) (17, 22). The COS is to standardize the minimum outcomes that should be reported in trials by consensus to reduce the heterogeneity in the selection of outcomes in similar clinical trials, and make the outcomes more consistent and comparable, thus helping resource integration and improving research value (20, 23). Because the COS is mainly used in efficacy outcomes, safety outcomes were always developed as one outcome in the COS, naming safety outcome, adverse event, or adverse reaction (23). The COS studies rarely reported how to report adverse events or what should be reported (24), and only a few studies reported both the COS of efficacy and safety (23).

When designing trials in the cardiovascular diabetology field, it is necessary to report enough but not too many outcomes for both safety and efficacy (25). Thus, it is very important to develop a COS that considers both the efficacy and safety outcomes to standardize the outcomes and reduce reporting bias (18, 26). So that it can help to integrate the results of multiple studies, which will benefit the systematic review and provide high-quality results for the diagnosis, treatment, and prevention of cardiovascular diabetology (27).

When developing a COS, we should follow the COS-STAR guidelines (28), the COS-STAP guidelines, and the handbook for developing COS. As the outcomes in cardiovascular diabetology are complex (12–14), potential modification of the steps will be performed based on the characteristics of cardiovascular diabetology in order to develop a high-quality COS. In the current paper, we report the methodological framework and hope it will help other researchers in this field.

## 2 Methods

### 2.1 The main aspects of the methodological framework

**Figure 1:**
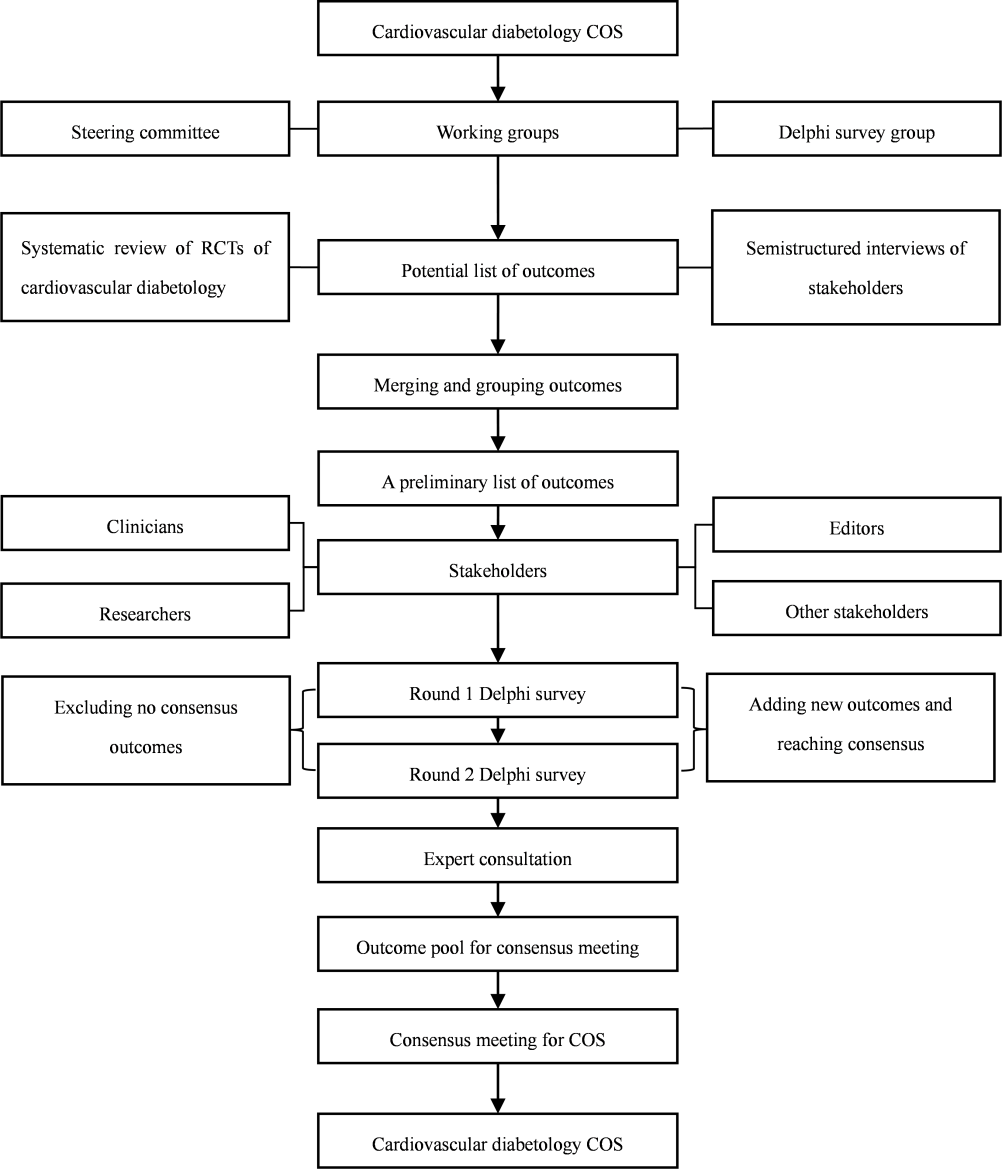
The development process for cardiovascular diabetology COS.

The cardiovascular diabetology COS will be developed following the COS-STAR guidelines (28) and it will be modified based on the characteristics of cardiovascular diabetology. The COS will be registered on the COMET website, and the details of the study will be shared online. The ethical approval for developing the cardiovascular diabetology COS has been obtained from the West China Hospital of Sichuan University (2023-502). Since the current methodological framework does not involve patients, it does not need to be approved by the ethics committee (29). **Figure 1** shows the development process of cardiovascular diabetology COS.

### 2.2 Establish COS groups of stakeholders

Three COS groups will be established with different functions (30, 31): (a) international steering committee will be established, the authors will invite expert in the field in China, and will also invite one or two international steering members from the USA, and one member of COS in UK to guide the study, the author will invite them via email and conference; the role of the group will be responsible for systematic review, guiding the semistructured interview process, planning, implementing, and organizing the development of consensus meetings; (b) Delphi survey group: the group will be included experts of clinicians, researchers, and nurses in cardiovascular diabetology field, the author will also invite editors of international journals involved cardiovascular diabetology, the author will invite them via email and conference; the role of the group will be as follows: review the outcomes, and select potential core outcomes; and (c) the consensus meeting group: the group will be included experts of clinicians, researchers, nurses and editors in cardiovascular diabetology field, the author will invite them via email and conference; the role of the group will be as follows: vote on the core outcomes at the consensus meeting, finally determine the COS.

#### 2.2.1 Inclusion criteria of international steering committee

(a) Clinicians (18, 22): with a bachelor’s degree or above, engaged in clinical research and related work on cardiovascular diabetology for more than 10 years, and with senior professional titles; (b) researchers (18): with outstanding academic achievements, he/she has published papers related to clinical field of cardiovascular diabetes as the first or contact author. The international steering committee will propose five people to be responsible for systematic review, guide the semistructured interview process, and plan, implement, and organize the development of consensus meetings.

#### 2.2.2 Inclusion criteria of the Delphi Survey Group

(a) Clinicians (18, 22): with a bachelor’s degree or above, engaged in clinical research and related work on cardiovascular diabetology for more than 10 years, and with senior professional titles;
(b) Researchers (18): with outstanding academic achievements, he/she has published papers related to the clinical field of cardiovascular diabetes as the first or contact author;
(c) Editors: editors who have worked in international journals involved in cardiovascular diabetology for more than five years, and edited at least 50 published trials of cardiovascular diabetology. The members of the Delphi survey group will be invited by sending invitations to international and national organizations involved in cardiovascular diabetology.

#### 2.2.3 Inclusion criteria of the International Steering Committee

The consensus meeting group will include all members of the expert steering group, other relevant clinicians, methodological experts, and editors. The number of consensus meeting groups will include about 20 experts.

### 2.3 Systematic review

Two authors will independently perform a systematic review of potential outcomes (22). The search databases will include PubMed, EMbase, CNKI, WanFang Data, and VIP database. The search time will be limited from 1 January 2021 to 31 December 2022. The inclusion criteria of the systematic review will be as follows (18, 26): (a) randomized controlled trial, cohort study, systematic review/meta-analysis; (b) the study should involve clinical aspects of diabetes/cardiovascular interrelationship and the metabolic syndrome; (c) outcomes should be reported; (d) study should be in Chinese or English; (e) studied subjects should be over 18 years old, and the sample size should be more than 50. The exclusion criteria of the systematic review will be as follows: (a) the full text is not available; (b) significant contradiction between the study content and conclusions. The search terms will include cardiovascular, diabetes, and trial, and will be modified with the characteristics of different databases. The search strategy for searching PubMed is shown in **Box 1**.

Box 1 **The search strategy for PubMed for searching the outcome of cardiovascular diabetology.**

#1 Diabetes Mellitus[Mesh] OR diabet*

#2 Mortality[Mesh] OR mortality OR Death[Mesh] OR death OR Survival[Mesh] OR survival OR Hospitalization[Mesh] OR hospital* OR utilization OR Cardiovascular Diseases[Mesh] OR cardiovascular diseases OR Myocardial Infarction[Mesh] OR myocardial infarction OR Angina Pectoris[Mesh] OR angina pectoris OR Coronary Disease[Mesh] OR coronary artery disease OR coronary heart disease OR Acute Coronary Syndrome[Mesh] OR acute coronary syndrome OR Heart Failure[Mesh] OR heart failure OR cerebrovascular events

#3 Systematic review OR meta-analysis OR RCT OR randomized controlled trial OR cohort study

#4 #1 AND #2 AND #3

Two researchers will independently read the titles and abstracts. Uncertain or potentially qualified literature will be decided after the full-text screening. The researchers will independently extract the data, including the first author, time of publication, title, study characteristics, and outcomes. Inconsistent results will be discussed or consulted with by the third researcher. All outcomes from these studies will be used to form the outcome pool.

### 2.4 Semistructured interview

The semistructured interviewees will invite 30 stakeholders who are willing to participate in the interview. According to previous studies (18, 22, 26), the 30 interviewees will reach saturation. At least one patient will be interviewed to include patient perspectives. Systematically trained professional graduate students will interview stakeholders face-to-face after signing the informed consent form (18, 22, 26). Each interview will be conducted for about 20–30 min. The interview will include the basic information of the interviewees (name, gender, age, contact information), interview time, and interview outline content. Each interview will include three open questions: What do you think are the top 5 outcomes that need to be reported in cardiovascular diabetology? Are these outcomes easily measured? How will you sort these outcomes?

All interviews will be audio recorded and transcribed verbatim (27), and transcripts will be imported into NVivo software. All transcripts will be anonymized. The interviews will be coded using the principles of thematic content analysis (26). Relevant outcomes will be identified and appropriately coded from the transcripts using a provisional coding framework based on the outcomes extracted from the systematic review (27). The new outcomes proposed in the interviews will be added to the outcome pool after being identified by the steering committee.

### 2.5 Determine the original outcome item pool

The outcomes from a systematic review and semistructured interview will be combined to establish the item pool (18). Appropriate combinations will be performed when the outcomes are expressed by the same or similar means. The item pool will be reviewed and approved by the steering committee.

### 2.6 Delphi survey

#### 2.6.1 Delphi survey questionnaire

The Delphi survey questionnaire’s content includes the participants’ general information (educational background, major, age, gender, and work) (22). The language of the questionnaire will be Chinese and English. The original list of outcomes will be arranged according to the classifications to form the questionnaire, and scoring items will be set under each outcome (18). The scoring criteria will be referred to by the Likert scoring method: 1–3 points will be dis-important, 4–6 points will be important, and 7–9 points will be very important (18, 26). At the end of the questionnaire, a blank item will be set, and participants will be asked to add the outcomes that still need to be included (18).

#### 2.6.2 Delphi survey

The Delphi survey will be conducted by electronic questionnaire (18). It will be conducted in two rounds. The second round will be adjusted according to the first round (22, 30). In the first round, the questionnaire will be sent to experts, who will complete the survey within 2 weeks. A reminder email will be sent at the end of the first week, and at the end of 2 weeks, statistical analysis will be performed on the results of the first survey round (18). The results of the first round will be included: (a) general content of establishment of the COS for clinical studies: number of questionnaires sent, number of questionnaires recovered, number of participants completed, and proportion of questionnaires completed; (b) scoring content: the scoring results and distribution of each outcome (including the mean, minimum, and maximum values of scores and the consensus of different scores), the outcomes with an average score of 6 points or above will be ranked according to the first round from high to low, and the second round of questionnaires will be sent to all the experts who completed the first round of the survey (22, 25).

The experts will also be asked to re-score each item and explain if necessary (if the experts change the score from dis-important or important in the first round (less than 6) to very important in the second round (greater than or equal to 6) or from very important in the first round to important or dis-important in the second round, to be noted) (22, 25, 29). At the same time, the number of respondents and the distribution of scores in the first round of the survey will be fed back to the experts participating in the second round of the survey in the form of a histogram (22, 25, 29). The items in the content survey results will be reported in the same way as in the first round after the second round.

After completing the second round of the survey, the author will screen the outcomes with an average score of more than 6 points and the outcomes with total scores as supplementary outcomes and sort out the reasons given by the experts (18, 26). A summary of the distribution of the numbers and scores of all participants in the survey will be made (18, 26). In addition, the changes in participants’ scores in two rounds of surveys will be analyzed and summarized.

### 2.7 Expert consultation

In order to get more detailed information, expert consultation will be performed after analyzing the results of the Delphi survey. The invited experts will be five clinicians, one methodological expert, and one editor. They will discuss the results of the Delphi survey and decide the potential outcomes that will be included in the outcome pool for the expert consensus meeting. Finally, the outcome pool for the expert consensus meeting will be formed.

### 2.8 The expert consensus meeting

The expert consensus meeting will be held after the expert consultation (18, 26). The contents of the consensus meeting will be as follows: (a) report the research methods and results of the outcome pool of cardiovascular diabetology; (b) discuss the outcomes with and without consensus according to the research results and recommend the final core outcomes; and (c) discuss whether outcome measure tools and measure time can be recommended according to the outcome to be included in the COS. The consensus standards are shown in **Table 1** (29). Potential disagreements among the experts or between the experts and other stakeholders will be resolved by discussion or voting. After the consensus meeting, the cardiovascular diabetology COS will be formatted. Experts are then invited to promote the application of the core outcome sets.

**Table 1:** Consensus definition of cardiovascular diabetology COS (29).

Consensus in: consensus that the outcome should be included in the core outcome set	Uncertainty about the importance of the outcome	Consensus out: consensus that the outcome should not be included in the core outcome set.
70% or more participants scoring as 7–9 and fewer than 15% participants scoring as 1–3	Any other scoring	50% or fewer participants scoring it 7–9

## 3 Discussion

In clinical trials, the inconsistent selection of outcomes is common, which leads to the fact that many clinical trials cannot be compared in the same category or combined in the systematic review/meta-analysis (17), thus reducing the value of trials and wasting resources. The COMET Initiative was established to promote the production of the smallest outcome set, namely the COS, which should be reported in clinical trials, and promote the improvement of methodology and international communication and cooperation for research on the COS by integrating resources for research (32). At present, more than 200 working groups have completed the study of the COS, which provides a powerful methodological reference for constructing the COS. Although there is currently no “gold standard” for constructing the core outcome set, the COMET Manual, COS-STAR statement, and COS-STAD recommendations can provide essential reference values for the current study of the COS.

The current methodological framework presents the processes of developing a COS in cardiovascular diabetology. The problems of outcomes in cardiovascular diabetology will be reviewed. Based on the experience of constructing a COS, the construction method of the COS for clinical research in cardiovascular diabetology will be proposed, and the method’s feasibility will also be verified. This future study will support completing the first COS for clinical trials in cardiovascular diabetology. In addition, we will try our best to disseminate it with the help of the stakeholders, and we also hope the future COS will be published in a high-impact journal so that it can be accepted and adopted by the broader scientific and medical community.

Nonstandard diagnostic criteria for diseases, nonstandard names of outcomes, unclear definitions of outcomes, a lack of outcome measurement tools, and multiple measurement time points for the same outcome always exist in trials (20, 29). Thus, developing COS is crucial for the research field of cardiovascular diabetology because it will help improve the methodology of trials (30). Ensuring consistent outcome reporting will reduce reporting bias, improve data synthesis and comparison, and enable better clinical interpretation and application of the current evidence base (29). A minimum number of critical outcomes will be reported in future clinical studies through developing the COS for cardiovascular diabetology, with which clinicians and stakeholders worldwide agree. It will help strengthen the current evidence base in cardiovascular diabetology through standardized reporting.

## Data availability statement

The original contributions presented in the study are included in the article/supplementary material. Further inquiries can be directed to the corresponding authors.

## Author contributions

JJ: Writing – original draft. LC: Writing – original draft. YP: Writing – review & editing, Supervision. QJ: Writing – review & editing, Methodology, Validation. YH: Writing – review & editing, Validation. YZ: Writing – review & editing. NL: Writing – review & editing, Supervision, Validation.

## References

[1] Di Mario C Genovese S Lanza GA Mannucci E Marenzi G Sciatti E . Role of continuous glucose monitoring in diabetic patients at high cardiovascular risk: an expert-based multidisciplinary Delphi consensus. Cardiovasc Diabetol (2022) 21(1):164. doi: 10.21203/rs.3.rs-1446808/v1 36030229 PMC9420264

[2] Drożdż K Nabrdalik K Kwiendacz H Hendel M Olejarz A Tomasik A . Risk factors for cardiovascular disease in patients with metabolic-associated fatty liver disease: a machine learning approach. Cardiovasc Diabetol (2022) 21(1):240. doi: 10.1186/s12933-022-01672-9 36371249 PMC9655870

[3] Conti CR . Cardiovascular diabetology. Clin Cardiol (1999) 22(11):685–6. doi: 10.1002/clc.4960221102 PMC6656223 10554681

[4] Thomas MC Coughlan MT Cooper ME . The postprandial actions of GLP-1 receptor agonists: The missing link for cardiovascular and kidney protection in type 2 diabetes. Cell Metab (2023) 35(2):253–73. doi: 10.1016/j.cmet.2023.01.004 36754019

[5] Di Pietrantonio N Di Tomo P Mandatori D Formoso G Pandolfi A . Diabetes and its cardiovascular complications: potential role of the acetyltransferase p300. Cells (2023) 12(3):431. doi: 10.3390/cells12030431 36766773 PMC9914144

[6] Shah BR Austin PC Ke C Lipscombe LL Weisman A Booth GL . Growing income-related disparities in cardiovascular hospitalizations among people with diabetes, 1995 to 2019: population-based study. Diabetes Care (2023) 46(4):751–6. doi: 10.2337/figshare.21870582.v1 36720121

[7] Dar MS Wanner C Marx N Ofstad AP Mattheus M Kaspers S . Cardiovascular outcomes trial data from EMPA-REG OUTCOME, CAROLINA, and CARMELINA: assessment of a novel staging system for type 2 diabetes. Diabetes Obes Metab (2023) 25(5):1372–84. doi: 10.1111/dom.14989 36700391

[8] Hu MJ Hu S Tan JS Yang YJ . Individual or familial diabetes in relation to eight cardiovascular diseases: A two-sample Mendelian randomization study. Nutr Metab Cardiovasc Dis (2023) 33(4):883–91. doi: 10.1016/j.numecd.2023.01.018 36775708

[9] Davis TME Giczewska A Lokhnygina Y Mentz RJ Sattar N Holman RR . Effect of race on cardiometabolic responses to once-weekly exenatide: insights from the Exenatide Study of Cardiovascular Event Lowering (EXSCEL). Cardiovasc Diabetol (2022) 21(1):116. doi: 10.1186/s12933-022-01555-z 35761271 PMC9238154

[10] Leccisotti L Cinti F Sorice GP D'Amario D Lorusso M Guzzardi MA . Dapagliflozin improves myocardial flow reserve in patients with type 2 diabetes: the DAPAHEART Trial: a preliminary report. Cardiovasc Diabetol (2022) 21(1):173. doi: 10.1186/s12933-022-01607-4 36057768 PMC9440459

[11] Liu Y Bharmal SH Kimita W Petrov MS . Effect of acute ketosis on lipid profile in prediabetes: findings from a cross-over randomized controlled trial. Cardiovasc Diabetol (2022) 21(1):138. doi: 10.1186/s12933-022-01571-z 35871064 PMC9308353

[12] Sasso FC Simeon V Galiero R Caturano A De Nicola L Chiodini P . The number of risk factors not at target is associated with cardiovascular risk in a type 2 diabetic population with albuminuria in primary cardiovascular prevention. Post-hoc Anal NID-2 trial. Cardiovasc Diabetol (2022) 21(1):235. doi: 10.1186/s12933-022-01674-7 PMC9641842 36344978

[13] Tanaka A Imai T Shimabukuro M Taguchi I Sezai A Toyoda S . Association between serum insulin levels and heart failure-related parameters in patients with type 2 diabetes and heart failure treated with canagliflozin: a *post-hoc* analysis of the randomized CANDLE trial. Cardiovasc Diabetol (2022) 21(1):151. doi: 10.1186/s12933-022-01589-3 35941584 PMC9358857

[14] Simeone P Tripaldi R Michelsen A Ueland T Liani R Ciotti S . Effects of liraglutide vs. lifestyle changes on soluble suppression of tumorigenesis-2 (sST2) and galectin-3 in obese subjects with prediabetes or type 2 diabetes after comparable weight loss. Cardiovasc Diabetol (2022) 21(1):36. doi: 10.1186/s12933-022-01469-w 35277168 PMC8917620

[15] Hoogeboom TJ Jette AM . Using evidence hierarchies to find the best evidence: A procrustean bed? Phys Ther (2021) 101(11):pzab235. doi: 10.1093/ptj/pzab235 34865127

[16] Munn Z Stern C Aromataris E Lockwood C Jordan Z . What kind of systematic review should I conduct? A proposed typology and guidance for systematic reviewers in the medical and health sciences. BMC Med Res Methodol (2018) 18(1):5. doi: 10.1186/s12874-017-0468-4 29316881 PMC5761190

[17] Convie LJ Clements JM McCain S Campbell J Kirk SJ Clarke M . Development of a core outcome set for informed consent for therapy: An international key stakeholder consensus study. BMC Med Ethics (2022) 23(1):79. doi: 10.1186/s12910-022-00820-w 35945581 PMC9364552

[18] Sheng X Chen C Ji Z Hu H Zhang M Wang H . Development of a core outcome set on Traditional Chinese Medicine and Western Medicine for rheumatic heart disease: a study protocol. BMJ Open (2022) 12(11):e062497. doi: 10.1136/bmjopen-2022-062497 PMC9660565 36368756

[19] Munblit D Nicholson T Akrami A Apfelbacher C Chen J De Groote W . A core outcome set for post-COVID-19 condition in adults for use in clinical practice and research: an international Delphi consensus study. Lancet Respir Med (2022) 10(7):715–24. doi: 10.1016/S2213-2600(22)00169-2 PMC9197249 35714658

[20] Kirkham JJ Gorst S Altman DG Blazeby JM Clarke M Tunis S . Core outcome set-STAndardised protocol items: the COS-STAP statement. Trials (2019) 20(1):116. doi: 10.1186/s13063-019-3230-x 30744706 PMC6371434

[21] van Hunsel F de Waal S Härmark L . The contribution of direct patient reported ADRs to drug safety signals in the Netherlands from 2010 to 2015. Pharmacoepidemiol Drug Saf (2017) 26(8):977–83. doi: 10.1002/pds.4236 28524293

[22] Jia M Lu Y Liang X Tong C Wang J Tang J . Development of a core outcome set for hypertensive intracerebral hemorrhage in clinical trials of traditional Chinese medicine: a study protocol. Trials (2022) 23(1):871. doi: 10.1186/s13063-022-06801-z 36224599 PMC9559838

[23] Lei R Shen Q Yang B Hou T Liu H Luo X . Core outcome sets in child health: A systematic review. JAMA Pediatr (2022) 176(11):1131–41. doi: 10.1001/jamapediatrics.2022.3181 36094597

[24] Liu JP Han M Li XX Mu YJ Lewith G Wang YY . et al: Prospective registration, bias risk and outcome-reporting bias in randomised clinical trials of traditional Chinese medicine: an empirical methodological study. BMJ Open (2013) 3(7):e002968. doi: 10.1136/bmjopen-2013-002968 PMC3717464 23864210

[25] Qiu R Zhong C Wan S Zhang Y Wei X Li M . Developing a core outcome set for assessing clinical safety outcomes of cardiovascular diseases in clinical trials of integrated traditional Chinese medicine and Western medicine: study protocol. Trials (2022) 23(1):239. doi: 10.1186/s13063-022-06166-3 35346338 PMC8962576

[26] Dai XY Zi MJ Liu CX Wang YM Gao R . Development of a core outcome set in the clinical trials of traditional Chinese medicine for diabetic foot: A study protocol. Front Med (Lausanne) (2022) 9:1025833. doi: 10.3389/fmed.2022.1025833 36438030 PMC9682122

[27] Li G Han R Lin M Wen Z Chen X . Developing a core outcome set for clinical trials of chinese medicine for hyperlipidemia. Front Pharmacol (2022) 13:847101. doi: 10.3389/fphar.2022.847101 35586053 PMC9108338

[28] Kirkham JJ Gorst S Altman DG Blazeby JM Clarke M Devane D . Core outcome set-STAndards for reporting: the COS-STAR statement. PloS Med (2016) 13(10):e1002148. doi: 10.1371/journal.pmed.1002148 27755541 PMC5068732

[29] Xiao L Shi L Liu S Luo Y Tian J Zhang L . A core outcome set for clinical trials of first- and second-degree perineal tears prevention and treatment: a study protocol for a systematic review and a Delphi survey. Trials (2021) 22(1):843. doi: 10.1186/s13063-021-05820-6 34823584 PMC8614027

[30] Liu X Ma Q Chen J Yang H . A protocol for developing core outcome sets for laparoscopic hiatal hernia repair. Trials (2022) 23(1):907. doi: 10.1186/s13063-022-06845-1 36303243 PMC9612608

[31] Mitchell JW Noble A Baker G Batchelor R Brigo F Christensen J . Protocol for the development of an international Core Outcome Set for treatment trials in adults with epilepsy: the EPilepsy outcome Set for Effectiveness Trials Project (EPSET). Trials (2022) 23(1):943. doi: 10.1186/s13063-022-06729-4 36397081 PMC9670528

[32] Gargon E: . The COMET (Core outcome measures in effectiveness trials) initiative. Maturitas (2016) 91:91–2. doi: 10.1016/j.maturitas.2016.06.007 27451326

